# Dentinomimetics and cementomimetics of *Moringa oleifera* leaves extract

**DOI:** 10.1038/s41598-023-46656-1

**Published:** 2023-11-07

**Authors:** Raneem F. Obeid, Mohamed M. Ammar, Sara H. Younis

**Affiliations:** Faculty of Oral and Dental Medicine, Future University in Egypt, Cairo, 11835 Egypt

**Keywords:** Caries risk assessment, Fluoride varnish, Non-bonded restorations, Dental materials, Endodontics

## Abstract

To evaluate the biomimetic remineralization capabilities of *Moringa oleifera* leaves (MOL) extract on coronal dentin and acellular cementum, two different concentrations (50 and 200 mg/ml) of MOL extract loaded in plain varnish (M1 and M2 groups respectively) were compared to fluoride varnish (FL group) and native surface (C group). Eighty sound premolar teeth were collected. Forty teeth (10 teeth in each group) were used for coronal dentin testing while the other forty (10 teeth in each group) were used for acellular cementum testing. Teeth in M1, M2, and FL groups were etched for 30 s and then received the specific varnish treatment. All samples were immersed in artificial saliva for 14 days and then collected, dried, and examined by scanning electron microscopy and energy dispersive X-ray spectroscopy (EDX). Histologically, FL group showed mineral deposition as discrete vesicular granules of various sizes on the surface of both coronal dentin and acellular cementum. Mineral deposition only occurred on some DTs openings while opened tubules remained. The surface of the acellular cementum revealed regular grooves, micro-fissures, and cracks. In the M1 and M2 groups, mineral deposition appeared as a homogenous continuous layer on coronal dentin and acellular cementum. Only a few DTs and cementum fissures were not filled completely. In L.S. sections of the coronal one-third, the DTs appeared almost sealed with varying lengths of mineral deposition. EDX results statistical analysis showed that the M2 group had the highest phosphate ions (P^−^) and calcium ions (Ca^+2^) at%. MOL has an extraordinary effect on the remineralization of coronal dentin and acellular cementum. It would have a promising ability to control dentinal hypersensitivity and formation of biomimetic cementum tissue.

## Introduction

The awareness towards the use of nature increased the holistic medical practices in different therapies including the oral healthcare profession. Nowadays, “herbal” medicine is growing very fast^1^. *Moringa oleifera* (MO) is considered a leafy green legumes that is rich in many beneficial elements such as β-carotene, vitamin C^2^, many minerals such as Calcium which is more than what is present in milk (2185 mg/100 g in dry leaves), Phosphorus (252 mg/100 g in dry leaves), Potassium (1236 mg/100 g in dry leaves)^3^ and plant-based oxalates as a natural compound (oxalic acid)^4^ which are essential for medicinal use. They also have more protein than eggs, more iron than spinach, more vitamin A than carrots, carotenoids, flavonoids, and phenols^3,5^. The analysis of *Moringa oleifera* leaf (MOL) extract revealed that it contains 15 kinds of amino acids such as phenylalanine and tryptophan and non-essential amino acids including histidine, proline, glycine, arginine, serine, cysteine and aspartic acid^6^. Moringa leaves contains vitamin B complex, Copper, Potassium, Zinc, Silica, Magnesium, Manganese and Omega 3, 6^7^. It could be possible that the availability of all the previous elements promotes the different remineralization processes in human hard tissues^8^.

The complex human dentin is the major component of the human teeth that protects the pulp tissue. Dentin contains small canals along its structure called dentinal tubules (DTs) that are connected to the pulp tissues and is covered by either enamel or cementum^9^. Dentin hypersensitivity (DH) is a sharp pain that occurs after mechanical, chemical or thermal stimulation of bare coronal or root dentin due to loss of the protective enamel or cementum, respectively. Wide or patent DTs lead to movement of dentinal fluid inside these tubules, and this indirectly stimulates the nerve endings leading to hypersensitivity sensation. But fortunately, this hypersensitivity could be controlled if these DTs became completely or partially occluded^10^. Many agents have been used for management of DH either by controlling the flow of tubular fluid or by occluding the DTs^11^. For example, potassium salts show great effect on reducing DH when used in tooth pastes as potassium nitrate along with fluoride^12^.

Cementum is an extremely vulnerable tissue under the frequently-occurring diseases as the root caries due to its composition and hierarchical structure^13^. Moreover, its self-repairing capacity is limited and the repaired one is quite different from the physiological one^14^. Type I collagen and fluorine-containing nano-hydroxyapatite are the cementum predominant components^15^, and the content of fluorine is higher in cementum than in other mineralized hard tissues^16^. So it plays a special beneficial role as a biomineralizing catalyst in cementum remineralization accelerating the deposition of Ca^2+^ ion and leading to conversion of precursors to apatite^17^. This fluorine-containing nano-hydroxyapatite is responsible for the enhanced mechanical properties of cementum including hardness, elastic modulus, and toughness^15,18^*.* So, in order to promote cementum regeneration many therapeutic approaches (guided tissue regeneration and application of enamel matrix protein), have been conducted but their clinical outcomes are still far from perfect^19^. As mentioned by Gungormus et al.^20^, Cementomimetics is the construction of a cementum-like biomineralized microlayer via amelogenin-derived peptides. Amino acid sequences in natural proteins are responsible for mineral binding which facilitate the deposition of minerals on cementum surface and promote remineralization of surface lesions^20^.

It has been concluded by the authors in 2020 that MOL can enhance the rebuilding of enamel surface lesions, MOL minerals might be able to undergo chemical interaction with enamel minerals, and the peptide-guided remineralization might be the foundation for its ability to deposit a new layer that resembles the structure of healthy enamel^21^, But no studies till now have been found evaluating the biomimetic capabilities of MOL extract on cementum and dentin. So, the aim of the present study was to evaluate the power of MOL extract on blocking the DTs through the formation of protective barrier and depositing a new layer of cementum that resembles healthy cementum. The null hypothesis of this study states that MOL extract with two concentrations will have the same remineralization ability of the gold standard remineralizing agent (FL).

## Results

### Ultrastructure results of dentin and cementum (SEM)

#### Group C

**Figure 1:**
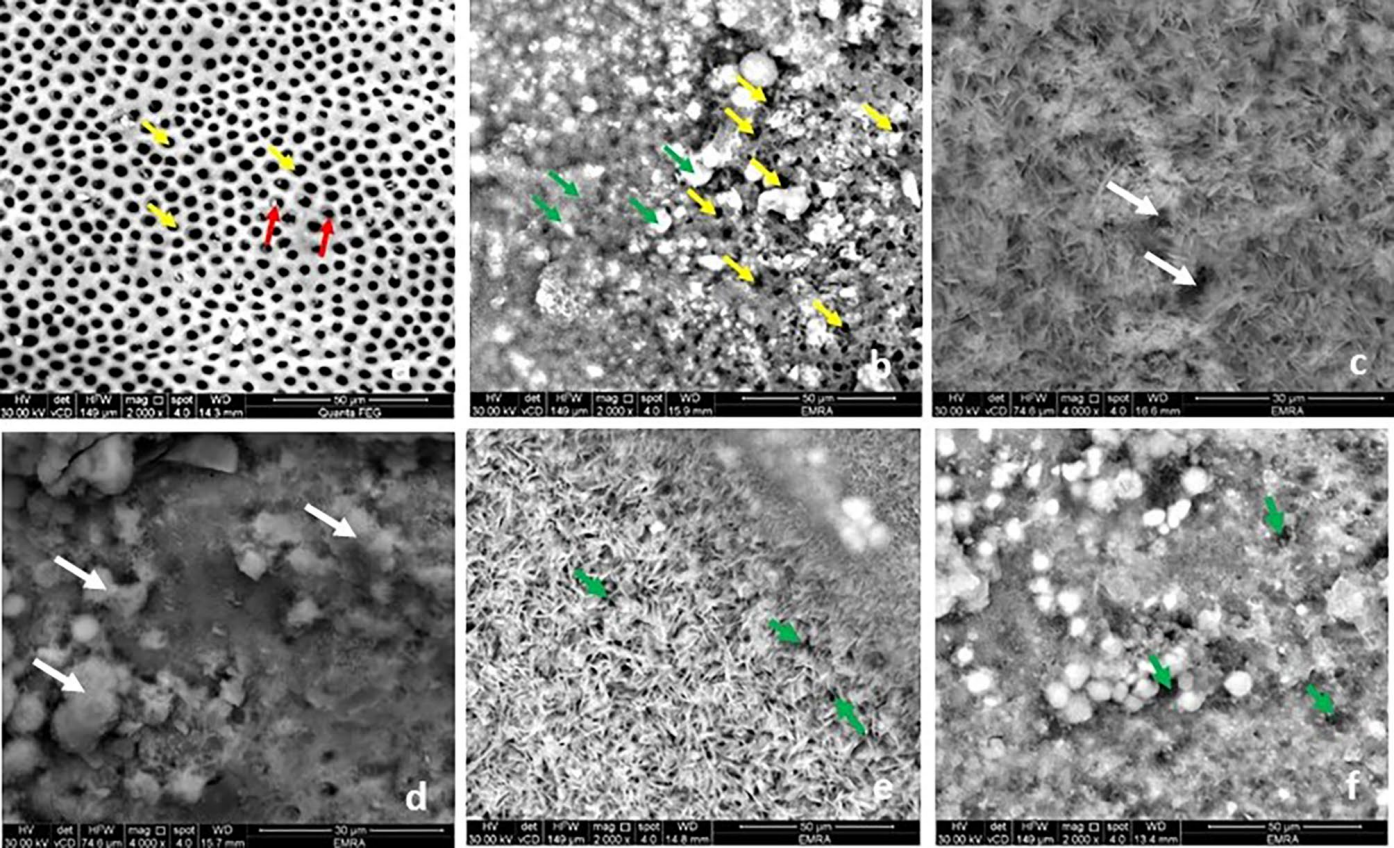
Scanning electron micrograph showing the DTs in the coronal dentin (T.S): group C, opened tubules (yellow arrows) with peritubular dentin surrounding it (red arrows) (**a**) (magnification × 2000); Group FL, Minerals deposition on the DTs opening in some areas (green arrows) and opened tubules in others (yellow arrows) (**b**) (magnification × 2000).; Group M1, some DTs are still evident opened (white arrows) (**c**,**d**) and two figures of minerals deposition; needle shaped crystals **(c)** and globular or vesicular shape (**d**) (magnification × 4000).; Group M2, dense meshwork of needle like hydroxyapatite crystals (**e**) and vesicular granules deposition (**f**). The DTs diminished dramatically (green arrows) (**e**,**f**) (magnification × 2000)**.**

When assessed by SEM control group showed normal configuration of coronal dentin and acellular cementum. The coronal dentin opened DTs with peritubular dentin encircling their boundaries (Fig. 1a). The acellular cementum appeared as a granular vesicular layer. It exhibited few grooves, micro fissures, and sporadic cracks giving its characteristic coral pattern (Fig. 3a).

#### Group FL

**Figure 2:**
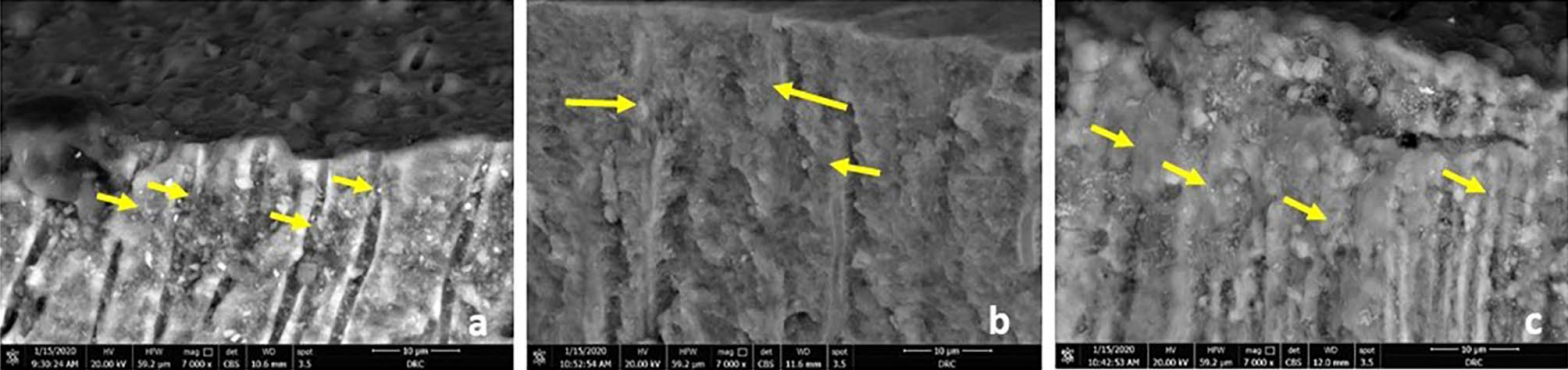
Scanning electron micrograph showing the DTs in the coronal dentin (L.S): group FL, scattered minerals deposition in the DTs (green arrows) (**a**); Group M1, minerals deposition in the DTs (yellow arrow) (**b**): Group M2 more minerals deposition in the DTs (yellow arrow) (**c**)) (magnification × 7000)**.**

**Figure 3:**
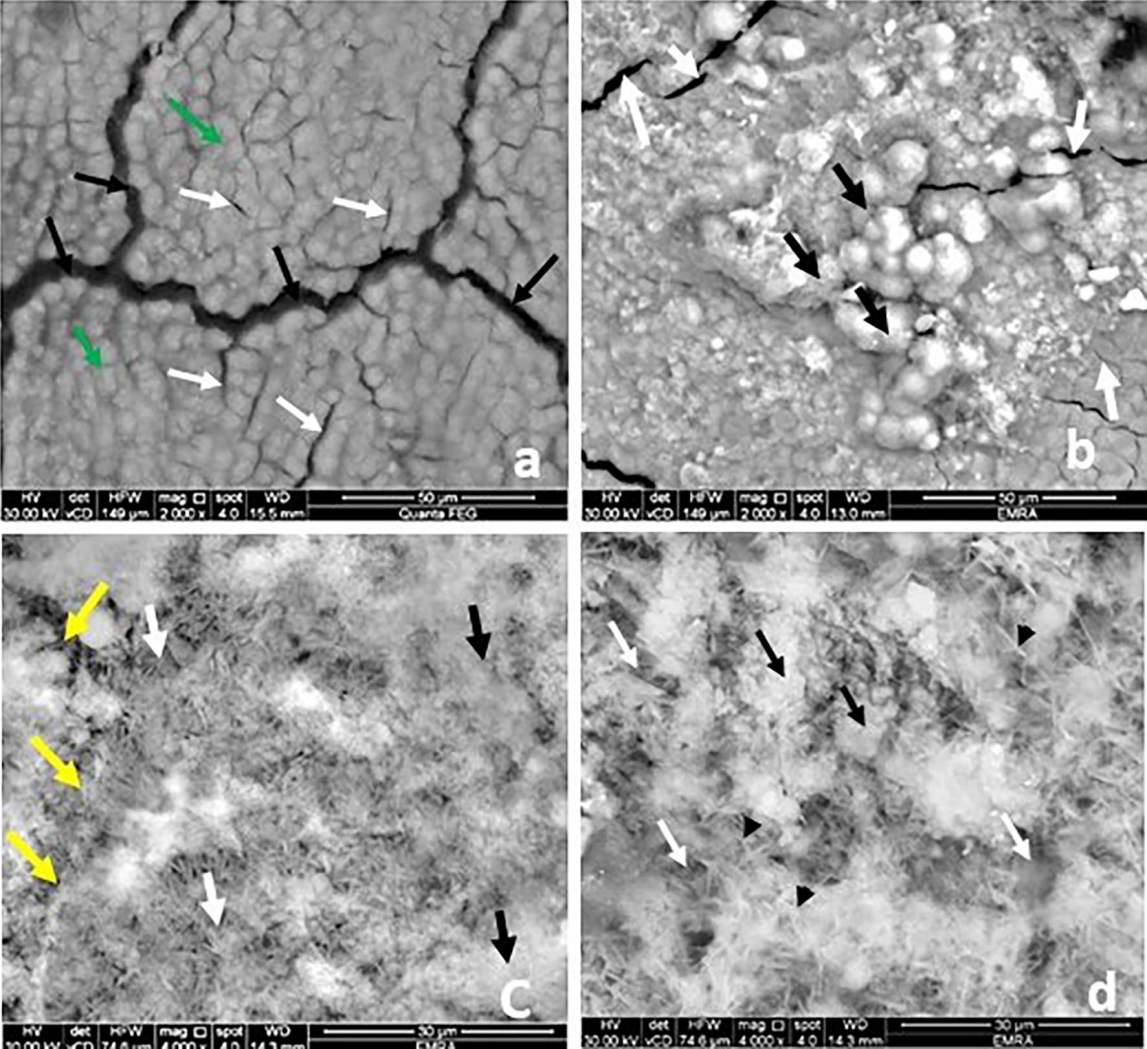
Scanning electron micrograph showing the acellular cementum: group C, few grooves (green arrows), micro fissures (white arrows) and cracks (black arrows) (**a**); Group FL, granular minerals deposition (black arrows), opened micro fissures and grooves (white arrows) (magnification × 2000) , (**b**); Group M1, a continuous layer of minerals deposition and fissures not filled completely with minerals (yellow arrows) and two figures of minerals deposition; needle shaped crystals (white arrows) and globular or vesicular shape (black arrows) (magnification × 2000) (**c**); Group M2, meshwork filled by needle like hydroxyapatite crystals (black arrows heads) and vesicular granules deposition (black arrows) (magnification × 4000). There are few fissures and grooves still opened (white arrows**) (d**) (magnification × 4000).

After fluoride varnish application, this group showed minerals deposition as vesicular granules of various sizes on the surface of both transverse section (T.S) and longitudinal section (L.S.) of coronal dentin and the acellular cementum. The DTs in T.S coronal dentin were evident, though many were obliterated by mineral granules (Fig. 1b). Also, in the L.S the DTs showed scattered mineral deposition with different levels of obliteration in some DTs (Fig. 2a). The surface of acellular cementum revealed the regular grooves, micro fissures, and cracks, yet to a lesser extent when compared to group C (Fig. 3b).

#### Group M1

In comparison to the group FL, group M1 examined by SEM showed more minerals deposition. It appeared as a homogenous continuous layer of minerals on the coronal dentin and the acellular cementum. Yet few fissures and DTs (T.S) were not filled completely with minerals (Fig. 1c,d). The minerals deposition exhibited two figures of configurations. One arranged in a meshwork filled by needle like hydroxyapatite crystals with variant length and direction, the other showed minerals depositions in a globular or vesicular fashion (Figs. 1c,d and 3c). By examination of L.S sections, the DTs of coronal one third appeared almost sealed with varying length of mineral deposition (Fig. 2b).

#### Group M2

In comparison to the previous groups, specimens of group M2 treated teeth the surface of the coronal dentin (T.S) was loaded with minerals, almost obliterating its DTs by needle like hydroxyapatite crystals (Fig. 1e) and vesicular granules deposition (Fig. 1f). And by L.S the DTs appeared filled by minerals which plug the DT along their entire length unlike the previous group (Fig. 2c).Furthermore, acellular cementum appeared entirely covered with mineral deposition which nearly completely filled grooves, micro fissures and cracks (Fig. 3d) and similar to the lower concentration the two configurations of minerals deposition were further evident (Fig. 3d).

### Elemental analysis (EDX results)

**Figure 4:**
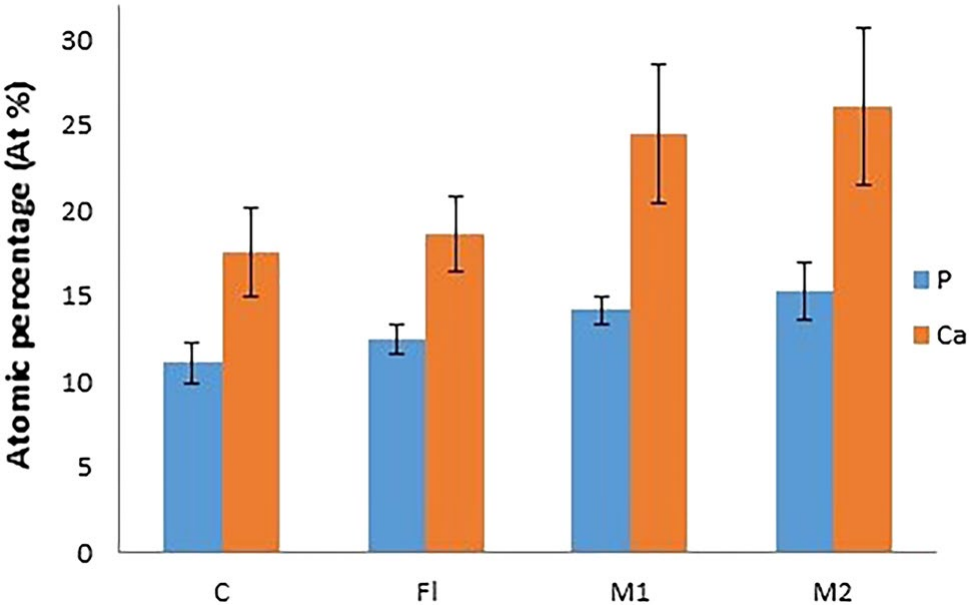
Mean and standard deviation representation of the at% of P^−^ and Ca^2+^ (surface elements) resulted from EDX analysis on coronal dentin surface.

**Figure 5:**
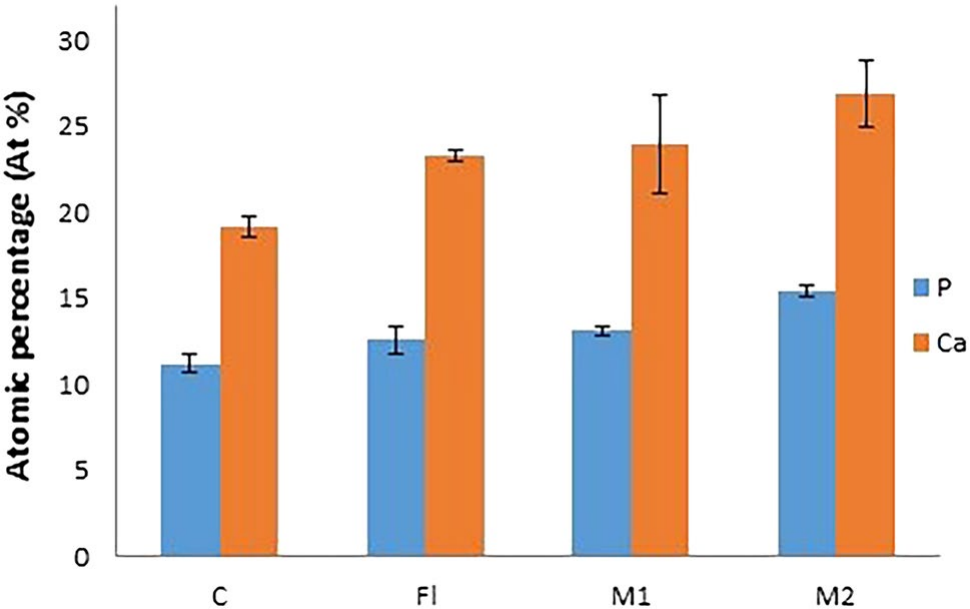
Mean and standard deviation representation of the atomic percentage (at%) of P^−^ and Ca^2+^ (surface elements) resulted from EDX analysis on acellular cementum surface.

EDX Spectra of investigated samples revealed that the main elements from the composition of the remineralized layer were P^−^ and Ca^2+^. Comparing the concentration of each element on the coronal dentin and acellular cementum after each treatment revealed differences between the groups (Figs. 4 and 5). The mean atomic percentage (at%) of each element between the study groups was compared using one way analysis of variance (ANOVA) test with Bonferroni posthoc. Two-sided* p* values less than 0.05 was considered statistically significant.

#### Mineral content within coronal dentin after different treatment protocols (Table 1 and Fig. 4)

**Table 1:** Mean, standard deviation (± SD) and one-way ANOVA with Bonferroni posthoc analysis of surface elements at% on coronal dentin surface (T.S).

DENTIN	C	FL	M1	M2	F	*p* value
P^−^	11.1 ± 1.2^a^	12.5 ± 0.9^a^	14.2 ± 0.8^b^	15.3 ± 1.7^b^	7.8	< 0.004
Ca^+2^	17.6 ± 2.6^a^	18.6 ± 2.2^a^	24.5 ± 4.1^b^	26.1 ± 4.6^b^	5.2	< 0.018

*Similar superscript letters mean no significant difference (*p* < 0.05).

For P^−^ at%, groups M1 & M2 were significantly higher than groups C & FL. Groups M1 & M2 were not significantly different between one another. Regarding Ca^+2^ at%, groups M1 & M2 were significantly higher than groups C & FL. Groups M1 & M2 were not significantly different between one another.

#### Mineral content within acellular cementum after different treatment protocols (Table 2 and Fig. 5)

**Table 2:** Mean, standard deviation (± SD) and one-way ANOVA with Bonferroni posthoc analysis of surface elements at% on acellular cementum.

Cementum	C	FL	M1	M2	F	*p* value
P^−^	11.2 ± 0.5^a^	12.6 ± 0.8^ab^	13.1 ± 0.3^bc^	15.4 ± 0.3^d^	35.2	< 0.001
Ca^+2^	19.1 ± 0.6^a^	23.3 ± 0.4^ab^	24.0 ± 2.9^bc^	26.9 ± 1.9^d^	9.8	< 0.005

*Similar superscript letters mean no significant difference (*p* < 0.05).

For P^−^ at%, groups M1 & M2 were significantly higher than group C, M2 was significantly higher than group FL & M1. Regarding Ca^+2^ at%, groups M1 & M2 were significantly higher than group C, M2 was significantly higher than group FL & M1.

## Discussion

Remineralization is the repair process that occurs naturally for non-cavitated lesions and depends on the presence of the essential ions (P^−^, Ca^2+^ and FL) along with assistant proteins (as arginine) to rebuild subsurface hydroxyapatite crystals on the remaining old ones^22,23^. Fluoride is the gold standard for remineralization; it comes in many forms such as mouthwashes, toothpastes, fissure sealants, filling materials, or even chewing gums. However, concerns about its side effects (tooth discoloration, skeletal weakness, neurological problems, high blood pressure) and questionable effect on dentin remineralization, have forced the researchers to investigate other synthetic or natural materials that can obliterate the DTs and control DH^23–25^.

The need of certain ions to support phosphate and calcium mineralization of dentin matrix (mineral induction of dentin matrix: crystallization) is mandatory. Silica, magnesium, and silver enhanced dentin crystallization, silica even functioned as a stronger promoter of dentin matrix mineralization than fluoride^26^. Exposure of DTs leads to DH, so numerous studies have been conducted with different active ingredients (as strontium chloride, potassium nitrate, oxalates, bioactive glass, calcium hydroxyapatite, potassium bicarbonate, calcium hydroxide, silver nitrate) to examine their ability of obliterating the DTs openings and they showed variable capabilities of DH^27–30^.

MOL extract with its content of high levels of minerals and proteins provide the necessary alkaline environment for mineral deposition which leads to precipitation of P^−^ and Ca^2+^ on enamel and cementum surfaces and within the DTs^31^.

In a previous investigation by the authors^21^ the effect of MOL extract on enamel remineralization has been investigated, however dentin and cementum hard tissues have different structures and compositions with more organic content (collagenous content), with different rebuilding/remineralization mechanisms. So, in the present study, MOL extract due to its extraordinary composition was examined for its effect on remineralizing artificially demineralized dentin and cementum hard tissues and for allowing mineral deposition inside DTs. MOL extract was lyophilized, and the resulting powder was loaded in a non-fluoride containing plain varnish with two different loading concentrations. These MOL extract loaded varnishes were compared to fluoride-containing varnish. Varnish was used as a carrier for the MOL extract instead of immersion of the samples in the extract solution to develop a sustained effect on the surfaces^32^.

SEM examination of the FL group showed mineral deposition as vesicular granules of various sizes on the DTs openings of coronal dentin and on the surface of the acellular cementum (Figs. 1b and 3b). Although the lumens of some of the DTs in coronal dentin were evidently opened, many were obliterated by mineral deposition granules with different levels of obliteration showing a degree of remineralization with normal appearance of peritubular dentin. The surface of the acellular cementum still revealed regular grooves, micro-fissures, and cracks, yet to a lesser extent when compared to control group (Fig. 3b). On the other hand, SEM micrographs of both moringa groups (Figs. 1c–f and 3c,d) revealed more homogenous continuous layer of minerals on the coronal dentin and the acellular cementum with two configurations (needle-like hydroxy appetite crystals and globular or vesicular appearance) compared to FL group. In addition, increasing the concentration of moringa in M2 group showed that the number of opened DTs diminished dramatically, and cementum fissures almost disappeared. In the L.S sections, the DTs of coronal one-third appeared almost sealed (blocked) by minerals deposition with varying deposition lengths depending on the test group. Moringa groups showed more continuous mineral penetration inside the DTs when compared to fluoride group that showed scattered depositions (Fig. 2b,c). In addition, M2 group showed longer and denser mineral deposition denoting more effective blockage (obliteration) of the tubules (Fig. 2c). These findings were in agreement with the same ultrastructural configuration found by the authors in their previous study on the enamel surface^21^ that was mainly attributed to the high content of both macro-and micronutrients of MO leaves^23^.

Examining the tested surfaces with EDX, showed high concentrations of both P^−^ and Ca^2+^ ions after application of the MOL on dentin and cementum in comparison to FL group (Figs. 4 and 5). However, no significant difference in these ions’ concentration was revealed in coronal dentin with increasing the concentration of MOL (difference between groups M2 & M1) (Fig. 4). On the other hand, the EDX analysis results of the acellular cementum showed a statistical significant difference in the deposition of P^−^ and Ca^+2^ after increasing the concentration of MOL (200 mg/ml) (Fig. 5), this could be due to the structural difference and mineralization process of cementum tissue in comparison to dentin. The remineralization of cementum is associated with the exchange of minerals between the cementum surface and the surrounding environment (MOL), as was found with enamel.

MOL extract showed more promising remineralizing effect on dentin and cementum compared to FL through the more dense and homogenous deposition of both P^−^ and Ca^2+^ ions on these surfaces^21,25^ as reported by the SEM and EDX results. This was greatly attributed to that MOL is rich in both of these ions^33^. These findings were in agreement with a study performed by Khalaf et al.^25^ who concluded that the presence of P^−^ and Ca^2+^ in the MOL extract decreased the diameter of DTs. Also, Ali et al.^34^ stated that the moringa extract decreased the dentin permeability by showing lower dye penetration depth in coronal dentin due to the deposition of various elements such as P^−^ and Ca^2+^ and DT occlusion. Moreover, the presence of oxalate^35^, flavonoids and different kinds of amino acids^36,37^ in MOL could probably had a role in enhancing the occlusion of DTs which can eventually reduce the dentin permeability^38^. Many studies showed that a plug that is formed by the synergic play between arginine, calcium carbonate, and phosphate ions can effectively seal exposed DTs. This plug is resistant to normal pulpal pressures and to acid challenge, which reduces the dentinal fluid flow and sub-sequentially the dentinal hypersensitivity^6,36^. Gungormus et al.^20^, investigated the effect of peptide-guidance on reconstruction of acid etched cementum specimens, and their results showed how specific peptides sequences created a cementomimetic layer equivalent in its mechanical properties to natural cementum. Also, the aspartic acid and serine amino acids have a role in crystallization and stabilization of the mineral phase in dentin remineralization due to their strong electronegativity, they bind to hydroxyapatite and enhance P^−^ and Ca^2+^ precipitation^21^, they act as nucleation centers for crystal formation, and regulate crystal growth and morphology^8^. As MOL extract is rich in these amino acids, these results support the function for MOL extract in both dentin and cementum remineralization.

Additionally, the high content of proanthocyanidins and flavonoids (collagen stabilizers) in moringa that^37^, promote cementomimetics in a more favorable and significant process. As collagen type I that is present in the human cementum, forms cross-striated fibrils which act as a scaffold for mineral crystals formation when a remineralizing agent is used, So the preservation of this organic matrix helps in maintaining the right framework for mineralization^32^. They also induces the biological mineralization and maintain the structural integrity after the mineralization process^39^. Mohamed et al.^40^ evaluated the effect of proanthocyanidin in combination with tri-calcium phosphate and fluoride on artificial caries lesion in cementum, and found that the addition of proanthocyanidin to tri-calcium phosphate significantly reduced collagen degradation depth of artificial root caries lesion. Moreover, proanthocyanidin was found to have a role in acting as a scavenger for free radical which helps in absorption of different ions as Ca^2+^^36,37^.

Therefore, within the limitations of our study of having the same oral environment of temperature and pH fluctuation and presence of masticatory mechanical effects, this present study represent the ability to restore demineralized natural human cementum with a biomimetic one that highly resembles the extraordinary complex composition and the hierarchical structure of natural cementum by the application of MOL extract through preserving cementum matrix, together with moringa’s ability to control DH. The null hypothesis was accepted since statistical and histological outcomes of the study, revealed that the remineralization capabilities of MOL extract was not significantly different from that of FL. Further investigation to reveal the mineralization mechanism is required.

## Materials and methods

### Materials

#### Moringa extract preparation

After necessary permission was taken to collect “*Moringa oleifera*” leaves and use them in different medicinal preparations under the supervision of the National Research Center, Giza, Egypt. Voucher specimens were deposited in the herbarium of the National Research Centre; [voucher No. MO-522 (*Moringa oleifera* L.) Giza, Egypt. Plants were authenticated by Prof. Mostafa El-Missiry, professor of phytochemistry and plant systematics, National Research Centre. Experimental research and field studies on plants (either cultivated or wild), including the collection of plant material, complied with relevant institutional, national, and international guidelines and legislation". According to previously mentioned preparation method in Younis et al.^21^. MOL was collected (from National Research Center Gardens), dried and grinded after been carefully washed under running water for 15 min. The grinded leaves were then immersed in 80% ethyl alcohol for extract preparation and the alcohol was evaporated using a rotary evaporator at 45 °C under reduced pressure. The obtained extract was then dissolved in distilled water, frozen at − 80 °C and lyophilized. For every 100 g grinded leaves, 21 g lyophilized extract powder were obtained. The lyophilized powder was then loaded in a plain varnish obtained from GC Fuji, Aichi, Japan, in two concentrations (50 mg/ml and 200 mg/ml).

### Methods

#### Teeth selection

Eighty maxillary premolars extracted during orthodontic treatments were collected from the surgical department, faculty of oral and dental medicine, Future University in Egypt. Teeth were carefully cleaned by ultrasonic cavitron (Satallic 5600, France) to remove any calculus or stains on their surfaces and then inspected for any defects. All teeth used in this experiment were decay free. Teeth were then divided equally according to tooth tissue (dentin or cementum) to be examined. The teeth were kept in sterile saline until use.

#### Study design

**Figure 6:**
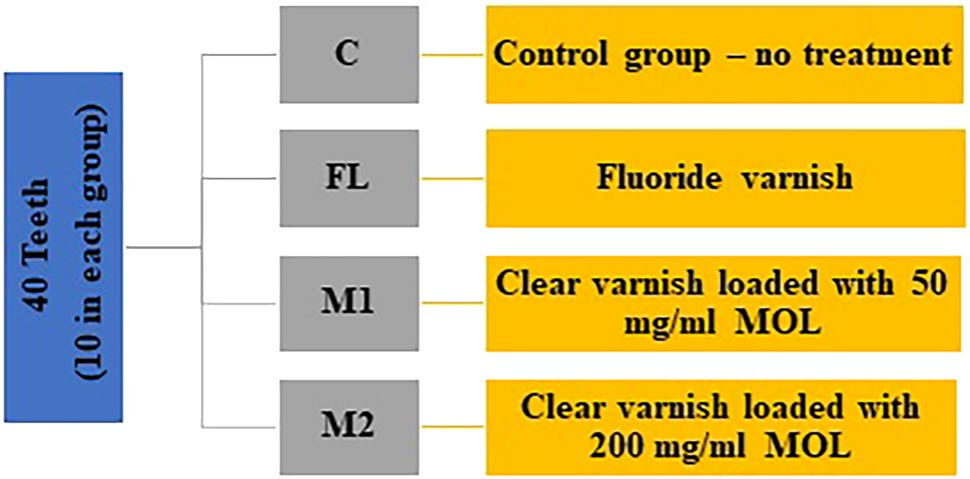
Experimental groups description.

For dentin surface treatment, teeth (n = 40) were prepared by cutting horizontally the occlusal third (the top 3 mm) to obtain a flat horizontal surface of dentin. For cementum surface treatment, a window was drawn by a liquid corrector pen in the cervical third of the buccal side of the root of each tooth (n = 40). The teeth of each treatment group were randomly equally assigned to four groups: Control (C), Fluoride (FL), MOL concentration 1 (M1) and MOL concentration 2 (M2). Teeth were gently cleaned by water spray for 10 s, air dried for 10 s and then an acid gel was applied for 20 s (phosphoric acid 37%, Jade, USA) on the area to be treated (full flat dentin surface or cementum window) (Fig. 6).

Each tooth in groups FL, M1 and M2 was then treated with fluoride varnish (Dharma Research, Florida, USA), 50 mg/ml moringa varnish and 200 mg/ml moringa varnish respectively. All teeth were immersed in artificial saliva solution prepared according to saporeti et al.^33^ in separate containers according to group and incubated at 37 °C for 14 days. Then, teeth were removed washed carefully with distilled water, air-dried and kept in a desiccator until examined. After the examination of coronal part of dentin, the teeth were sectioned parallel to the long axis of the tooth using a low-speed diamond disc^41,42^ to investigate the longitudinal section of it.

#### Scanning electron microscope/energy dispersive X-ray examination (SEM/EDX)

Teeth specimens from each group were scanned by SEM attached with EDX Unit (Quanta 250 FEG microscope, Netherlands) for dentin and cementum surfaces morphological microstructure and elemental composition changes. Representative microphotographs were captured, and elemental analysis of each specimen of each tissue was done.

#### Statistical analysis

Sample size calculation was done using 0.05 alpha level of significance (α) and 0.8 power of the study. The calculation was performed using PS “Power and Sample Size Calculations” software (version 3.1.6) for MS Windows. According to the results of previous studies^43,44^, a total sample size of 10 (per each group) was sufficient and an additional 10 samples in control group to be able to reject the null hypothesis.

Data were statistically described in terms of mean ± standard deviation (± SD). Numerical data were tested for the normal assumption using Shapiro Wilk test. Comparison of numerical variables between the study groups was done using one way analysis of variance (ANOVA) test with Bonferroni posthoc multiple 2-group comparisons. Two-sided* p* values less than 0.05 was considered statistically significant. IBM SPSS (Statistical Package for the Social Science; IBM Corp, Armonk, NY, USA) release 22 for Microsoft Windows was used for all statistical analyses.

### Ethical approval

The study design was approval by the Research Ethics Committee at Faculty of Oral and Dental Medicine, Future University in Egypt (FUE.REC (38)/12-2022) in accordance with the ethical standards of the national research committee.

#### Informed consent

For this type of study, formal consent is not required.

#### Institutional review board approval

The IRB of this study was approved by the Research Ethics Committee at Faculty of Oral and Dental Medicine, Future University in Egypt (FUE.REC (38)/12-2022). In our institution, a comprehensive consent is obtained when a patient’s teeth, are extracted in the OMS part. It states that the extracted teeth might be used for research in the future. Therefore, the individual patient’s consent is not required in the studies using only previously extracted teeth. Due to the retrospective nature of the study, the need of informed consent was waived by the Research Ethics Committee at Faculty of Oral and Dental Medicine, Future University in Egypt (FUE.REC (38)/12-2022)).

## Conclusion

MOL has an extraordinary effect when it comes to remineralization of dentin and cementum, it has a promising ability on controlling dentinal hypersensitivity and forming biomimetic cementum tissue. Clinical trials using MOL to control hypersensitivity sensation under deep restorative preparations and with root exposure will be of great dental value.

## Data Availability

The datasets used during the current study, available from the corresponding author on reasonable request.

## References

[1] Janakiram C, Venkitachalam R, Fontelo P, Iafolla TJ, Dye BA (2020). Effectiveness of herbal oral care products in reducing dental plaque and gingivitis—A systematic review and meta-analysis. BMC Complement. Med. Ther..

[2] Fozia F, Meenu R, Avinash T, Abdul AK, Shaila F (2012). Medicinal properties of *Moringa*
*oleifera*: An overview of promising healer. J. Med. Plants Res..

[3] Islam Z (2021). *Moringa*
*oleifera* is a prominent source of nutrients with potential health benefits. Int. J. Food Sci..

[4] Illingworth KA, Lee YY, Siow LF (2022). The effect of isolation techniques on the physicochemical properties of *Moringa*
*oleifera* protein isolates. Food Chem. Adv..

[5] El-sharkawy RT, El-kammar HA, Obeid RF, Bdelkhalek AA (2018). Effects of *Moringa*
*oleifera* aqueous leaf extract on submandibular salivary glands of diabetic albino rats. Egypt. Dent. J..

[6] Natsir H, Wahab AW, Budi P, Dali S, Arif AR (2019). Amino acid and mineral composition of *Moringa*
*oleivera* leaves extract and its bioactivity as antioxidant. J. Phys. Conf. Ser..

[7] Moringa—A serious superfood for healthy hair and skin. https://organicandwholesale.com/moringa-a-serious-superfood-for-healthy-hair-skin/.

[8] Zhang Y, Wang Z, Jiang T, Wang Y (2019). Biomimetic regulation of dentine remineralization by amino acid in vitro. Dent. Mater..

[9] Chiang Y-C (2016). A mesoporous biomaterial for biomimetic crystallization in dentinal tubules without impairing the bonding of a self-etch resin to dentin. J. Formos. Med. Assoc..

[10] Shiau HJ (2012). Dentin hypersensitivity. J. Evid. Based Dent. Pract..

[11] Miglani S, Aggarwal V, Ahuja B (2010). Dentin hypersensitivity: Recent trends in management. J. Conserv. Dent..

[12] Chu C-H, Lo EC-M (2010). Dentin hypersensitivity: A review. Hong Kong Dent J.

[13] Li X (2019). Severe periodontitis may influence cementum and dental pulp through inflammation, oxidative stress, and apoptosis. J. Periodontol..

[14] Chen K-Y (2009). Asymmetric chitosan membrane containing collagen I nanospheres for skin tissue engineering. Biomacromolecules.

[15] Yang T (2020). The construction of biomimetic cementum through a combination of bioskiving and fluorine-containing biomineralization. Front. Bioeng. Biotechnol..

[16] Tsuboi S (2000). Magnesium and fluoride distribution in human cementum with age. Calcif. Tissue Int..

[17] Feroz, S. & Khan, A. S. Fluoride-substituted hydroxyapatite. In *Handbook of Ionic Substituted Hydroxyapatites* 175–196 (Elsevier, 2020).

[18] Dawood AE (2018). Biocompatibility and osteogenic/calcification potential of casein phosphopeptide-amorphous calcium phosphate fluoride. J. Endod..

[19] Wei L (2019). Periodontal regeneration using bone morphogenetic protein 2 incorporated biomimetic calcium phosphate in conjunction with barrier membrane: A pre-clinical study in dogs. J. Clin. Periodontol..

[20] Gungormus M (2012). Cementomimetics—Constructing a cementum-like biomineralized microlayer via amelogenin-derived peptides. Int. J. Oral Sci..

[21] Younis SH, Obeid RF, Ammar MM (2020). Subsurface enamel remineralization by Lyophilized Moringa leaf extract loaded varnish. Heliyon.

[22] Featherstone JDB (2008). Dental caries: A dynamic disease process. Aust. Dent. J..

[23] Sujatha BK, Patel P (2017). *Moringa*
*oleifera*–Nature’s gold. Imp. J. Interdiscip. Res..

[24] Arifa MK, Ephraim R, Rajamani T (2019). Recent advances in dental hard tissue remineralization: A review of literature. Int. J. Clin. Pediatr. Dent..

[25] Nagib M, Amin L, Khalaf E (2016). Biological effects of topical applications of *Moringa*
*oleifera* extract versus fluoride on uremic patients extracted teeth. Int. J. Adv. Res..

[26] Saito T, Toyooka H, Ito S, Crenshaw MA (2003). In vitro study of remineralization of dentin: Effects of ions on mineral induction by decalcified dentin matrix. Caries Res..

[27] de Freitas SAA (2021). Bioactive toothpastes in dentin hypersensitivity treatment: A systematic review. Saudi Dent. J..

[28] da Silva ARJ, Deschamps Muniz RP, Almeida Lago MC, da Silva Júnior EP, Braz R (2022). Clinical efficacy of mouthwashes with potassium salts in the treatment of dentinal hypersensitivity: A systematic review and meta-analysis. Oper. Dent..

[29] Baghaban-Eslaminejad M, Oryan A, Kamali A, Moshiri A, Andronescu E, Grumezescu AM (2017). Chapter 25—The role of nanomedicine, nanotechnology, and nanostructures on oral bone healing, modeling, and remodeling. Nanostructures for Oral Medicine.

[30] Pałka ŁR (2020). In vitro SEM analysis of desensitizing agents and experimental hydroxyapatite-based composition effectiveness in occluding dentin tubules. Adv. Clin. Exp. Med. Off. Organ Wroclaw Med. Univ..

[31] Cummins D (2009). Dentin hypersensitivity: From diagnosis to a breakthrough therapy for everyday sensitivity relief. J. Clin. Dent..

[32] Pavan S, Xie Q, Hara AT, Bedran-Russo AK (2011). Biomimetic approach for root caries prevention using a proanthocyanidin-rich agent. Caries Res..

[33] Saporeti MP, Mazzieiro ET, Sales WF (2012). In vitro corrosion of metallic orthodontic brackets: influence of artificial saliva with and without fluorides. Dent. J. Orthod..

[34] Ali M-AS, Niazy MA, El-Yassaky MA (2021). Effect of application of natural versus synthetic desensitizing agents on dentin permeability. Al-Azhar Dent. J. Girls.

[35] Davari AR, Ataei E, Assarzadeh H (2013). Dentin hypersensitivity: Etiology, diagnosis and treatment; a literature review. J. Dent..

[36] Rahiotis C, Vougiouklakis G (2007). Effect of a CPP-ACP agent on the demineralization and remineralization of dentine in vitro. J. Dent..

[37] Epasinghe DJ, Yiu CKY, Burrow MF (2016). Effect of flavonoids on remineralization of artificial root caries. Aust. Dent. J..

[38] Hirsiger C (2019). Efficacy of 8% arginine on dentin hypersensitivity: A multicenter clinical trial in 273 patients over 24 weeks. J. Dent..

[39] Christner P, Robinson P, Clark CC (1977). A preliminary characterization of human cementum collagen. Calcif. Tissue Res..

[40] Mohamed AAA, El-Zainy MA, Rateb DGM (2020). Comparative study on the effect of grape seed extract and sodium fluoride on demineralized cementum of human premolar samples. Ain Shams Dent. J..

[41] Obeid M, Elshaboury E, Obeid R (2022). A comparative SEM assessment for the ability of PIPS, XP-Finisher and PUI to eliminate smear layer and open dentinal tubules. Egypt. Dent. J..

[42] Moustafa NM, Afifi R, Niazy MA (2020). Comparative evaluation for the effect of green tea, aloe vera and chlorhexidine on dentin erosion. Futur. Dent. J..

[43] El-Zainy MA, Halawa AM, Rabea AA (2012). The effect of some carbonated beverages on enamel of human premolars (scanning and light microscopic study). J. Am. Sci..

[44] Abd-Elmonsif NM, El-Zainy MA, Abd-Elhamid MM (2017). Comparative study of the possible effect of bovine and some plant-based milk on cola-induced enamel erosion on extracted human mandibular first premolar (scanning electron microscope and X-ray microanalysis evaluation). Futur. Dent. J..

